# Plasticity-Related Gene 5 Is Expressed in a Late Phase of Neurodifferentiation After Neuronal Cell-Fate Determination

**DOI:** 10.3389/fncel.2022.797588

**Published:** 2022-04-15

**Authors:** Isabel Gross, Nicola Brandt, Danara Vonk, Franziska Köper, Lars Wöhlbrand, Ralf Rabus, Martin Witt, Axel Heep, Torsten Plösch, Mark S. Hipp, Anja U. Bräuer

**Affiliations:** ^1^Research Group Anatomy, School of Medicine and Health Sciences, Carl von Ossietzky University of Oldenburg, Oldenburg, Germany; ^2^School of Medicine and Health Sciences, Carl von Ossietzky University of Oldenburg, Oldenburg, Germany; ^3^Department of Biomedical Sciences of Cells and Systems, University Medical Center Groningen, University of Groningen, Groningen, Netherlands; ^4^Perinatal Neurobiology Research Group, School of Medicine and Health Sciences, Carl von Ossietzky University of Oldenburg, Oldenburg, Germany; ^5^General and Molecular Microbiology, Institute for Chemistry and Biology of the Marine Environment (ICBM), Carl von Ossietzky University of Oldenburg, Oldenburg, Germany; ^6^Department of Anatomy, University Medical Center Rostock, Rostock, Germany; ^7^Research Center Neurosensory Science, Carl von Ossietzky University of Oldenburg, Oldenburg, Germany

**Keywords:** neurogenesis, PRGs, dendritic spines, neurodifferentiation, brain development

## Abstract

During adult neurogenesis, neuronal stem cells differentiate into mature neurons that are functionally integrated into the existing network. One hallmark during the late phase of this neurodifferentiation process is the formation of dendritic spines. These morphological specialized structures form the basis of most excitatory synapses in the brain, and are essential for neuronal communication. Additionally, dendritic spines are affected in neurological disorders, such as Alzheimer’s disease or schizophrenia. However, the mechanisms underlying spinogenesis, as well as spine pathologies, are poorly understood. Plasticity-related Gene 5 (PRG5), a neuronal transmembrane protein, has previously been linked to spinogenesis *in vitro*. Here, we analyze endogenous expression of the PRG5 protein in different mouse brain areas, as well as on a subcellular level. We found that native PRG5 is expressed dendritically, and in high abundance in areas characterized by their regenerative capacity, such as the hippocampus and the olfactory bulb. During adult neurogenesis, PRG5 is specifically expressed in a late phase after neuronal cell-fate determination associated with dendritic spine formation. On a subcellular level, we found PRG5 not to be localized at the postsynaptic density, but at the base of the synapse. In addition, we showed that PRG5-induced formation of membrane protrusions is independent from neuronal activity, supporting a possible role in the morphology and stabilization of spines.

## Introduction

Complex molecular processes during the development of the central nervous system (CNS) lead to an intricate network of highly specialized cells that communicate via synaptic activity. This development is established by a well-organized progression of events that are still poorly understood. Research has often focused on early embryonic stages of CNS development, but important steps of neuro-differentiation also continue after birth. Postnatal development involves axonal pathfinding and dendrite outgrowth, as well as establishing cell-cell contacts during spinogenesis and synaptogenesis (Lewis et al., 2013; Elston and Fujita, 2014). Even in adulthood, after development is completed, there are areas of the brain, retaining their capacity to generate new neurons, that then differentiate and are integrated into the existing network (Altman and Das, 1965; Paton and Nottebohm, 1984; Zhao et al., 2008; Ming and Song, 2011). In the mouse, this adult neurogenesis appears to occur only in the subventricular zone (svz) of the lateral ventricle and the subgranular zone (sgz) of the hippocampal dentate gyrus. Proliferating radial or non-radial precursor cells develop into transient amplifying cells in the svz, or into intermediate progenitor cells in the sgz, which can then both turn into neuroblasts. In the svz, doublecortin (DCX)-expressing neuroblasts migrate toward the olfactory bulb by forming a chain through a glial tube, called the rostral migratory stream (RMS). Upon arrival, they detach from the RMS by radial migration to the glomerular layer, where they differentiate and synaptic integration occurs (Lois et al., 1996; Lledo et al., 2006). In the second region of adult neurogenesis, the sgz in the hippocampus, the neuroblasts develop into immature neurons that start to migrate into the granule-cell layer and concurrently start to differentiate into dentate granule cells, including their synaptic integration into the hippocampal network (Lledo et al., 2006; Zhao et al., 2006; Ming and Song, 2011).

In both postnatal development and adult neurogenesis, late neurodifferentiation is characterized by the formation of dendritic spines (Stiles and Jernigan, 2010; Chen et al., 2014). These morphologically specialized structures extend from the dendrites and form the basis of most excitatory synapses in the brain. They exist in different sizes and shapes, and maintain a high capacity for plastic changes (Hering and Sheng, 2001; Tashiro and Yuste, 2003; Yuste and Bonhoeffer, 2004). Extensive research on dendritic spines reveals their importance for neuronal information processing, but the mechanisms of spine generation are still not well understood. Due to their central role in the functionality of the CNS, dendritic spines have been linked to several neurodegenerative pathologies, for example Alzheimer’s or Huntington’s disease. Additionally, proper spine development is impaired in neurodevelopmental disorders that are marked by intellectual disabilities, as in schizophrenia or autism-spectrum disorders (Herms and Dorostkar, 2016; Martinez-Cerdeno, 2017).

So far, several molecules involved in spinogenesis have been identified (Zhang and Benson, 2000; Sala et al., 2008). One of them is Plasticity-related Gene 5 (PRG5) a member of the lipid-phosphate phosphatase (LPP) superfamily (Coiro et al., 2014). LPPs are integral membrane proteins that consist of six transmembrane domains with three extracellular loops, containing one conserved ecto-phosphatase site (Brindley, 2004; Pyne et al., 2004; Sigal et al., 2005). By modifying their extracellular concentration or receptor affinity, they can modulate the signal transduction of lipid phosphate esters such as lysophosphatidic acid (LPA) or sphingosine-1-phosphate (S1P) (Brindley and Waggoner, 1998). In the LPP subfamily of PRGs, also called lipid-phosphate phosphatase related proteins (LPPRs), the conserved amino acids for the ecto-phosphatase activity have been modified. They therefore lack the enzymatic activity, but are still capable of modifying bioactive lipid signaling. The underlying mechanisms are still under investigation (Zhang et al., 2000; Brindley, 2004; Bräuer and Nitsch, 2008).

Another notable difference to the rest of the LPP family members is their vertebrate-specific and predominant neuronal expression (Bräuer et al., 2003). PRG5 (synonyms: LPPR5, PAP2D) mRNA is strongly expressed during mouse brain development, where it peaks around birth and slightly decreases toward adult stages. Only in areas of high synaptic plasticity and adult neurogenesis does PRG5 expression remain elevated (Coiro et al., 2014; Gross et al., 2021). *In vitro* analysis of developing hippocampal neurons showed a shift in expression, from an equal distribution along all neurites toward increased localization only along dendrites (Broggini et al., 2010; Coiro et al., 2014). The overexpression of PRG5 in immature primary hippocampal neurons led to an early increase in the formation of spine-like membrane protrusions. In mature neurons, PRG5 overexpression increased the number of homer-positive spines, as well as their spine-head diameters (Coiro et al., 2014). These results strongly support a functional role of PRG5 in dendritic spine development that should be analyzed during neuronal development.

Because previous PRG5 studies focused almost exclusively on *in vitro* experiments, the first aim of this study was to analyze endogenous PRG5 expression in mouse brain tissue. Therefore, we validated a specific PRG5 antibody and analyzed its protein detection in western blot experiments and with mass spectrometry. We performed immunofluorescence of different mouse brain regions and developmental stages. We found PRG5 to be ubiquitously expressed in the mouse brain, with high protein expression in regions of adult neurogenesis, such as the hippocampus and the olfactory bulb. However, we found PRG5 not to be involved in early processes of adult neurogenesis but associated with late post-mitotic neurodifferentiation. On a subcellular level, we found the PRG5 protein not to be located at the postsynaptic density and therefore postulate an important role of PRG5 in the formation and stabilization of dendritic spine morphology.

## Results

### Plasticity-Related Gene 5 Is a Glycosylated Membrane Protein in Mouse Brain Tissue

**FIGURE 1:**
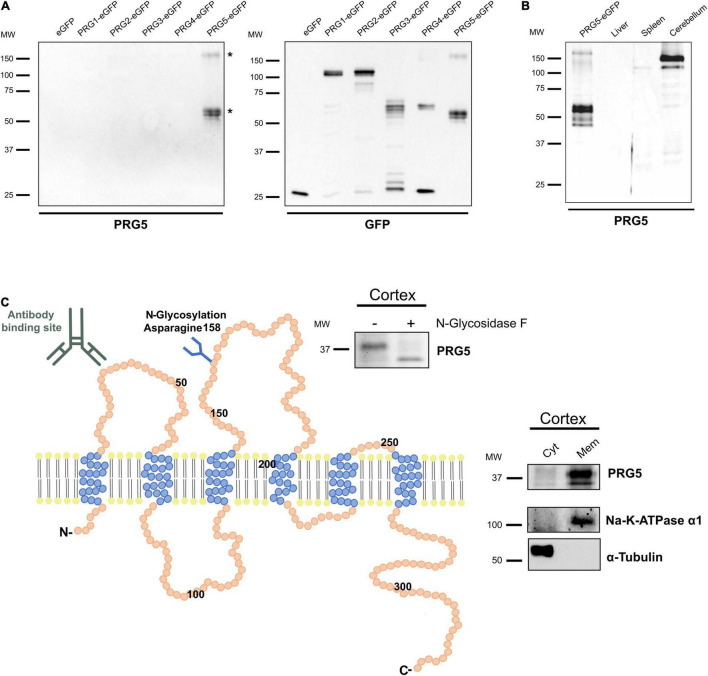
PRG5 is a glycosylated membrane protein in brain tissue. **(A)** Specificity analysis of a polyclonal PRG5 antibody shows specific binding only to PRG5 and no cross-reactivity to other PRG family members. Fusion proteins of PRG1-5 with eGFP were used for western blotting. The PRG5 antibody detected bands around 50 kDa and above 150 kDa. Bands marked with asterisks (*) were analyzed by MS and contained PRG5 peptides. **(B)** Western Blot analysis of protein lysates of adult mouse tissue shows strong PRG5 signal in the cerebellum, but not in liver or spleen (probed with anti-PRG5 antibody). Loading control is shown in Supplementary Figure 1. **(C)** Schematic depiction of the murine PRG5 protein and its orientation in the plasma membrane based on its amino acid sequence (primary accession number Q8BJ52). The six transmembrane regions are illustrated in blue, N- and C-termini are located intracellularly. The antibody-binding site in the first extracellular loop is indicated. An N-glycosylation site at asparagine 158 in the second extracellular loop is indicated, and was supported by western blot of P15 mouse cortex lysate treated with (+) and without (–) N-glycosidase F, and a subsequent shift of the PRG5 band to a lower molecular weight after treatment (blot above). Endogenous membrane localization of PRG5 was validated by western blot of cytosolic (Cyt) and membrane (Mem) protein fractions of P15 mouse cortex lysates (blot on the right). α-tubulin was used as a cytosolic marker, Na-K-ATPase α1 as a membrane marker. MW, molecular weight.

Existing studies of PRG5 expression were predominantly based on mRNA analysis, and protein studies so far focused primarily on *in vitro* experiments (Broggini et al., 2010; Coiro et al., 2014; Yu et al., 2015). To further investigate the role of PRG5 in the mouse brain, we identified a previously used PRG5 polyclonal antibody to be suitable for western blot and immunofluorescence in mouse brain tissue samples (Coiro et al., 2014). Antibody specificity to PRG5, compared to other PRG family members, was verified by western blotting of isolated eGFP constructs from HEK293H cells overexpressing all PRGs, and the respective detection of antibodies (Figure 1A). The antibody specifically detected the isolated PRG5-eGFP protein construct in bands at 60 kDa, equivalent to the predicted molecular weight of PRG5 and eGFP protein combined, but also in a band above 150 kDa. These bands were additionally analyzed by mass spectrometry (MS), and both contained PRG5 peptides (Figure 1A, bands marked with asterisks, for MS data see Supplementary Table 1). In a previous study, quantitative real-time PCR of different mouse tissue samples revealed highest PRG5 mRNA expression in the brain. Additionally, low mRNA expression in heart, lung, and testes was found, whereas no mRNA expression was detected in thymus, liver, spleen, and kidney (Coiro et al., 2014). We therefore used liver and spleen protein lysates as negative controls and cerebellum protein lysate as positive control for the anti-PRG5 antibody in western blot analysis. We found a strong PRG5 signal around 150 kDa and a weak signal around 37 kDa in cerebellum lysates. No PRG5 signal was detected in liver and spleen protein lysates (Figure 1B and Supplementary Figure 1).

Like its PRG family members, PRG5 is a six-transmembrane protein with intracellular N- and C-termini (Figure 1C; Brindley, 2004; Bräuer and Nitsch, 2008; Strauss and Bräuer, 2013). The antibody binding site in the first extracellular loop is indicated in Figure 1C. We previously demonstrated membranous protein expression of PRG5 in protein lysates of cultured primary hippocampal neurons (Coiro et al., 2014). Here, we confirm this membrane localization of PRG5 for brain tissue by analyzing postnatal day 15 (P15) mouse cortex lysates (Figure 1C). We also demonstrated N-glycosylation of the native PRG5 protein in mouse cortex lysate by a band shift after hydrolyzation of N-linked glycan chains using N-glycosidase F (Figure 1C).

### The Plasticity-Related Gene 5 Protein Is Developmentally Regulated in the Mouse Cortex

**FIGURE 2:**
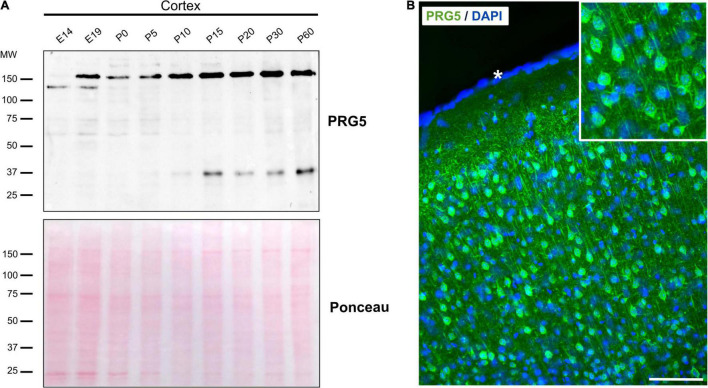
PRG5 protein expression in the mouse cortex increases during maturation. **(A)** Representative western blot analysis of total protein lysates of mouse cortex tissue between E14 and P60, probed with an anti-PRG5 antibody. The 150 kDa band was detected from E19 and increased toward adult stages. The 37 kDa band was detectable from P10. Only a very weak band was detectable in younger stages. Ponceau-S staining of total proteins is shown as a loading control. A representative blot of three technical repeats for each of three tissue preparations is shown. MW, molecular weight. **(B)** Immunostaining of PRG5 (green) and DAPI (blue) in a sagittal section of P15 mouse cortex. PRG5 expression was detected in neurons of all cortical layers. Asterisk (*) indicates pial surface. Scale bar = 100 μm.

We previously analyzed PRG1-5 expression on the mRNA level during mouse brain development via quantitative RT-PCR and found expression patterns to be dynamic (Velmans et al., 2013; Coiro et al., 2014; Gross et al., 2021). Here, we confirm a developmental regulation also for the PRG5 protein in cortex lysates from developmental stages E14 to P60 (Figure 2A). A protein band around 150 kDa was observable from embryonic stages on, and increased toward adulthood. On the other hand, a 37 kDa band, equivalent to the predicted molecular weight of PRG5, was clearly detectable at P10 and its intensity remained stable in adult stages. At younger stages, this band showed weaker intensity. Changes of PRG5 protein level during development were also found in hippocampus, olfactory bulb, and cerebellum (Supplementary Figure 2A).

For the first time, we were able to analyze PRG5 protein localization in sagittal mouse brain sections using immunohistochemistry. Staining of adult cortex sections supported the western blot results, as we found strong PRG5 signal throughout all cortical layers. There, PRG5 was localized to the soma and to dendritic structures of cortical neurons (Figure 2B).

### Plasticity-Related Gene 5 Protein Is Mainly Dendritically Expressed in the Mouse Brain

**FIGURE 3:**
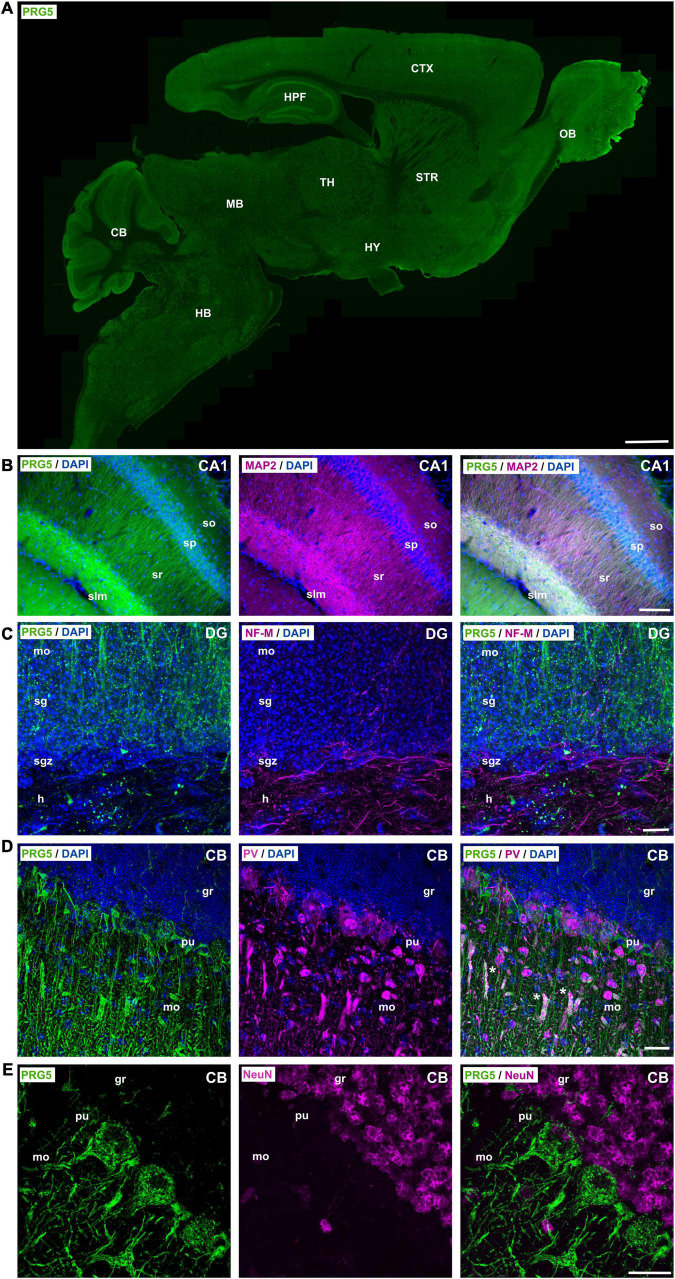
PRG5 is enriched in dendritic areas of the mouse hippocampus, olfactory bulb, and cerebellum. **(A)** Montage of a sagittal adult mouse brain section labeled with PRG5 (green) shows highest PRG5 expression in the cerebellum, hippocampus, and the olfactory bulb. Mosaic stitched from 635 tiles. Scale bar = 1 mm. **(B)** Dendritic expression of PRG5 (green) in the hippocampal CA1 region shown by co-immunostaining with MAP2 (magenta). PRG5 expression was found in the soma of pyramidal cells of the sp and increased in their dendritic arbors in the sr and slm. Scale bar = 100 μm. **(C)** Low axonal expression of PRG5 in the DG of the hippocampus is shown by co-immunostaining with axonal marker NF-M (magenta). Strong PRG5 expression was found in granule cells in the sg and their dendritic arbors in the mo, whereas there was only low expression in their mossy fibers in the hilum. Scale bar = 10 μm. **(D)** Purkinje cells of the CB showed strong PRG5 signal in their soma and in their dendritic arbors in the mo. Unspecific overlapping staining of blood vessels is marked with asterisks. Scale bar = 20 μm. **(E)** NeuN positive granule cells of the granule layer of the CB show no PRG5 expression. Scale bar = 20 μm. DAPI visualizes cell nuclei (blue). CA, cornu ammonis; CB, cerebellum; CTX, cerebral cortex; DG, dentate gyrus; gl, glomerular layer; gr, granule layer; h, hilus; HB, hindbrain; HPF, hippocampus formation; HY, hypothalamus; ipl, inner plexiform layer; MB, midbrain; mi, mitral layer; mo, molecular layer; MOB, main olfactory bulb; opl, outer plexiform layer; pu, Purkinje layer; sg, stratum granulosum; sgz, subgranular zone; slm, stratum lacunosum-moleculare; so, stratum oriens; sp, stratum pyramidale; sr, stratum radiale; STR, striatum; TH, thalamus. Representative images; Stainings of brain sections from at least three different animals were analyzed and revealed similar results.

After we demonstrated the presence of PRG5 protein in cortex lysates and sections, we aimed to analyze its specific localization in other brain areas. For this purpose, we performed immunohistochemical staining of adult (P30 and older) mouse-brain sagittal and coronal sections. We detected PRG5 immunofluorescence throughout the brain, but enriched in the hippocampal formation (HPF), cerebellum (CB), and the olfactory bulb (OB) (Figure 3A and Supplementary Figures 2B–D). Fluorescent signals of PRG5 were very prominent in dendritic regions of the hippocampus, such as the stratum radiale (sr) and the stratum lacunosum-moleculare (slm) of Ammon’s horn (CA). Dendritic expression of PRG5 was verified by co-localization with the dendritic cytoskeletal protein microtubule-associated protein 2 (MAP2). Additionally, somata of pyramidal cells of the stratum pyramidale (sp) showed PRG5 positive staining (Figure 3B). Axonal regions of the hippocampus on the other hand, were marked by neurofilament-M (NF-M) staining and showed only weak or no PRG5 signal. Somata of granule cells in the stratum granulosum (sg) of the DG showed a strong expression of PRG5 that extended into the basal dendrites of the molecular layer (mo) (Figure 3C and Supplementary Figure 2B). However, neuronal stem cells in the sgz lacked PRG5 expression and were only marked by DAPI staining (Figure 3C and Supplementary Figure 2B).

In the cerebellum, (CB) we found that Purkinje cells (pu), marked by parvalbumin (PV), strongly expressed PRG5 in their soma and in their intricately branched dendritic arbors in the molecular layer (mo) (Figure 3D and Supplementary Figure 2D). However, NeuN-positive granule cells in the granule layer (gr) of the CB showed no PRG5 signal (Figure 3E).

### Plasticity-Related Gene 5 Is Expressed in Inhibitory and Excitatory Neurons and in an Oligodendrocyte Subtype, but Not in Astrocytes

**FIGURE 4:**
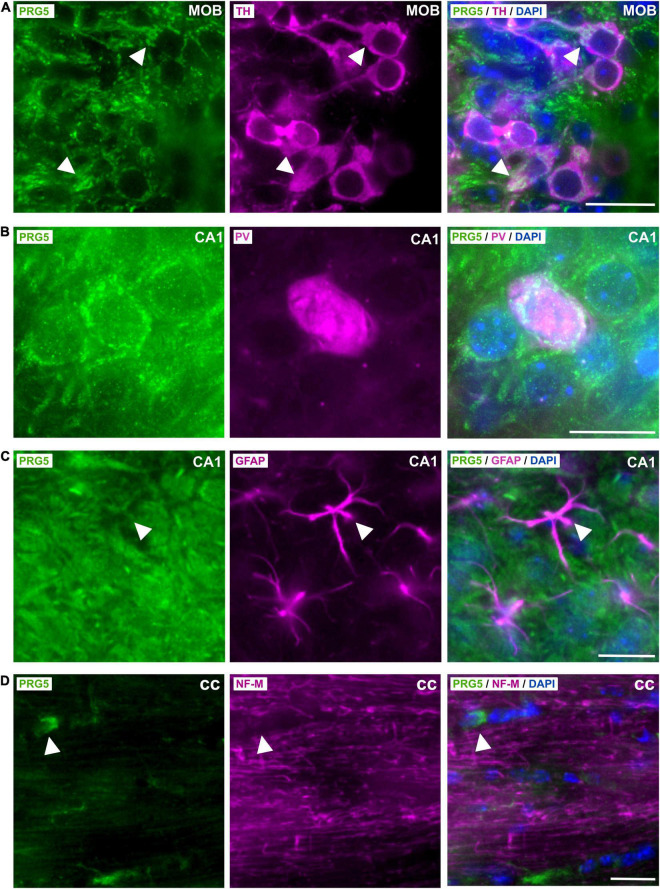
Cell-type specific expression of PRG5 in the brain. **(A)** TH-positive (magenta) dopaminergic neurons in the gl of the MOB expressed PRG5 (green). Scale bar = 20 μm. **(B)** PV (magenta)-positive inhibitory GABAergic interneurons in the hippocampus also expressed PRG5 (green). Scale bar = 20 μm. **(C)** PRG5 (green) was not expressed by GFAP-positive (magenta) astrocytes. Scale bar = 20 μm. **(D)** NF-M-expressing (magenta) axonal fibers in the cc did not show PRG5 (green) positive staining, but a PRG5-expressing subtype of oligodendrocytes was detected. Scale bar = 20 μm. DAPI was used to visualize cell nuclei (blue). CA, cornu ammonis; cc, corpus callosum; MOB, main olfactory bulb. Representative images; Stainings of brain sections from at least three different animals were analyzed and revealed similar results.

Tyrosine hydroxylase (TH) -positive dopaminergic neurons in the MOB expressed PRG5, as shown by an overlay of fluorescent signals (Figure 4A, white arrow heads). Co-staining against parvalbumin (PV) revealed PRG5 expression in inhibitory GABAergic interneurons, here shown in the CA1 region of the hippocampus (Figure 4B).

In line with previous *in vitro* results, where PRG5 mRNA expression was restricted to primary cultured neurons and was not found in primary cultured astrocytes (Coiro et al., 2014), we did not detect PRG5 in GFAP-positive astrocytes in the brain (Figure 4C, white arrow heads). On the other hand, we had previously demonstrated PRG5 mRNA expression in primary immature and mature cultured oligodendrocytes (Gross et al., 2021). In immunostaining of brain sections, we found PRG5 protein in a subtype of oligodendrocytes in the corpus callosum (cc), but we could not detect a PRG5 signal in axonal fibers of the cc, marked by NF-M (Figure 4D, white arrow heads).

### Plasticity-Related Gene 5 Is Widely Distributed in the Brain of Neonatal Mice (P0) but Not in Nestin-Positive Precursor Cells

**FIGURE 5:**
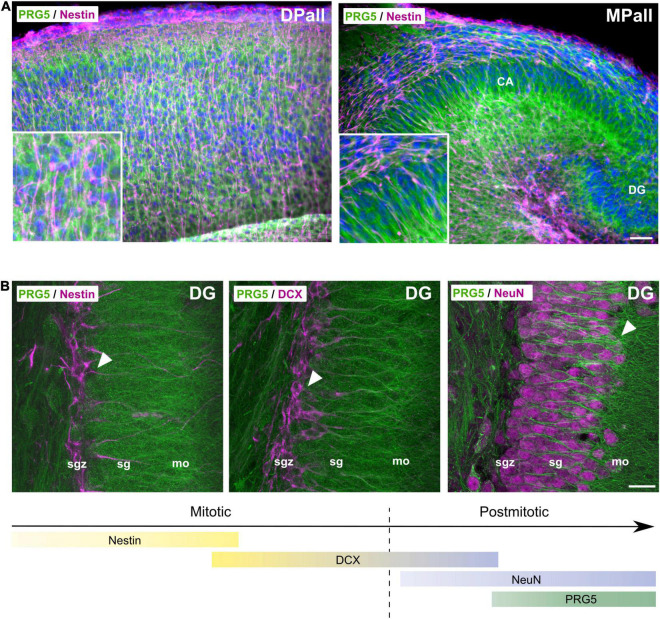
PRG5 protein is expressed after neuronal cell fate determination. **(A)** Representative sagittal sections of P0 mice brain, stained with antibodies against PRG5 (green) and nestin (magenta), revealed PRG5 expression in the developing isocortex (dorsal pallium, DPall) and the developing hippocampus (medial pallium, MPall) that was not colocalized to nestin-positive progenitor cells. Scale bar = 50 μm. **(B)** During adult neurogenesis in the sgz of the dentate gyrus in the hippocampus, PRG5 (green) expression was detected in a late postmitotic phase, together with NeuN (magenta, right image) expression, but not with mitotic neurodifferentiation markers nestin (magenta, left image) and DCX (magenta, middle image). Scale bar = 20 μm. CA, cornu ammonis; DG, dentate gyrus; DPall, dorsal pallium; mo, molecular layer, MPall, medial pallium; sg, stratum granulosum; sgz, subgranular zone. Representative images; Stainings of brain sections from at least three different animals were analyzed and revealed similar results.

After we identified a developmental regulation of PRG5 protein expression in the mouse brain, we analyzed neonatal mice brain (P0) sections for PRG5 expression, by immunostaining with the PRG5 antibody. We found a wide distribution of the PRG5 protein throughout all brain areas, with particularly high abundance in the medial pallidum (MPall), the developing hippocampus (Figure 5A). Neuronal and glial precursor cells, marked by a nestin antibody, did not show PRG5-positive staining (Figure 5A), indicating that the PRG5 protein is first expressed after neuronal cell fate has been determined.

### Plasticity-Related Gene 5 Protein Expression Is a Marker for Late Neurodifferentiation During Adult Neurogenesis

To gain further insight into the time point of PRG5 expression during neurodifferentiation, we analyzed its expression during adult neurogenesis. In Figure 5B, marker proteins of different developmental stages were used, together with PRG5 staining in the sgz of the dentate gyrus (DG) of the hippocampus. Early neurogenesis is marked by nestin and later by doublecortin (DCX) expression of mitotic immature neurons (Gleeson et al., 1999; Michalczyk and Ziman, 2005). Cells that are positive for either nestin or DCX, do not express the PRG5 protein (Figure 5B). During neuronal maturation, neurons downregulate DCX and increase their expression of NeuN, which is a marker for postmitotic neurons (Mullen et al., 1992; Gleeson et al., 1999). We found PRG5 protein abundance only in postmitotic NeuN-expressing neurons that had migrated from the sgz into the sg and the mo of the DG, indicating an involvement of PRG5 in late postmitotic neurodifferentiation processes (Figure 5B).

**FIGURE 6:**
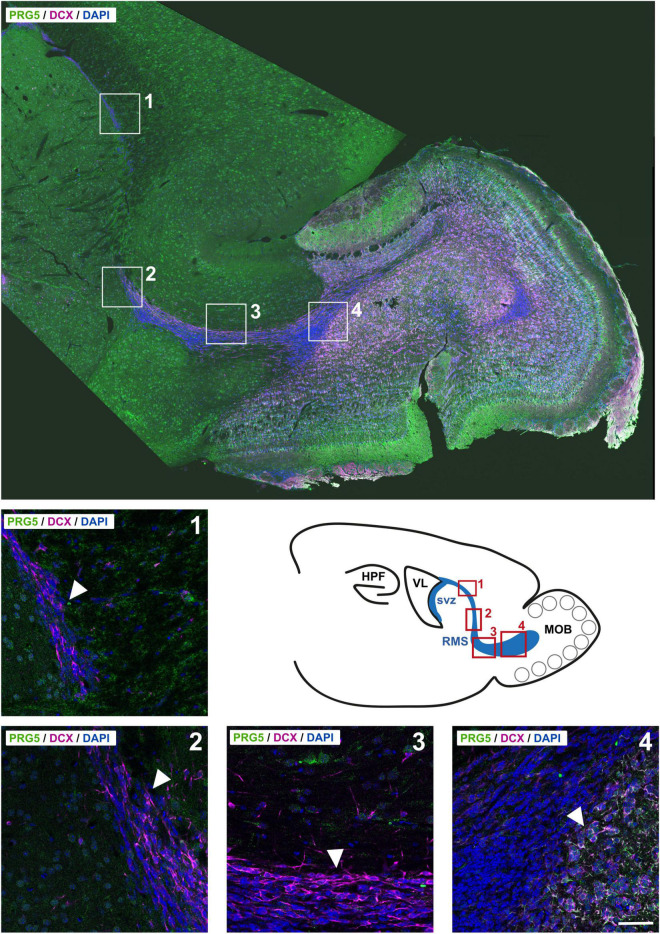
PRG5 protein expression is present in a late phase of neurodifferentiation in adult neurogenesis of the rostral migratory stream (RMS). Top: Montage of a sagittal adult mouse brain section labeled with PRG5 (green), DCX (magenta) and DAPI (cell nuclei, blue). Mosaic stitched from 234 tiles. Bottom: Full-resolution views of representative stages of migration in the RMS and schematic drawing of the svz, RMS, and the olfactory bulb in sagittal view. PRG5 protein abundance was only present at a late stage of neurodifferentiation, when neuroblasts entered the olfactory bulb and started differentiating. HPF, hippocampal formation; MOB, main olfactory bulb; RMS, rostral migratory stream, svz, subventricular zone; VL, lateral ventricle. Scale bar = 50 μm. Representative images; Stainings of brain sections from at least three different animals were analyzed and revealed similar results.

PRG5 protein abundance was not found during RMS migration of neuroblasts, but was first detected when they entered the olfactory bulb; it increased in radial migrating cells in the olfactory bulb (Figure 6). These findings suggest a specific role for PRG5 in late neuro-differentiation processes, rather than in neurogenesis itself.

### Plasticity-Related Gene 5 Is Not Localized at the Postsynaptic Density, but at the Base of the Synapse

**FIGURE 7:**
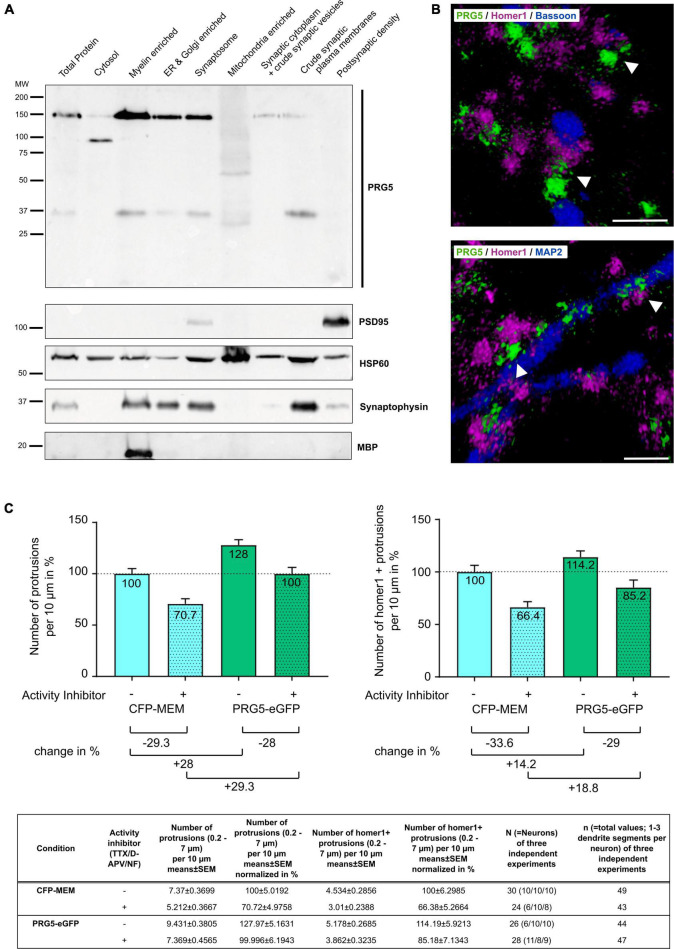
PRG5 stimulates formation of dendritic protrusions, independent of neuronal activity. **(A)** Representative western blot after subcellular fractionation of adult mouse cerebral cortex, probed with PRG5, with PSD95 as a postsynaptic density marker, HSP60 as a mitochondrial marker, synaptophysin as a synaptic marker, and MBP as a myelin marker. PRG5 was detected in myelin-, ER-, Golgi-enriched, and in synaptosomal fractions, predominantly around 150 kDa. The 37 kDa PRG5 band was mainly detected in the myelin-enriched and the crude synaptic plasma-membrane fractions. No PRG5 signal was found in the postsynaptic density fraction. A representative blot of three technical repeats for each of three tissue preparations is shown. MW, molecular weight. **(B)** Representative volume-rendered 3D projections of z-stack images of murine primary neurons cultured 15 days *in vitro* and stained for PRG5 (green), homer1 (magenta), and bassoon (top, blue) or MAP2 (bottom, blue). Stainings were analyzed for three independent neuron preparations. PRG5 protein was not localized to pre- and postsynaptic transmission sites, as shown by a lack of signal overlap to homer1 or bassoon proteins (top, white arrow heads). PRG5 was localized at the bases of postsynaptic spines, between postsynaptic density protein homer1 and dendritic-cytoskeletal protein MAP2 (bottom, white arrow heads). Scale bars = 1 μm. **(C)** Quantification of protrusions of mature primary neurons transfected either with CFP-MEM (control) or PRG5-eGFP with (+) and without (–) activity inhibition (1 μM TTX, 50 μM D-APV and 10 μM nifedipine). PRG5- overexpressing neurons showed elevated number of overall protrusions and of homer-positive protrusions compared to control cells after inhibition of neuronal activity. Number of protrusions was normalized to control (CFP-MEM) without activity inhibition (100%) and are shown as mean in% + SEM. Counted numbers and *n*-values are listed in the table below graphs. Statistical data is shown in Supplementary Table 2.

Another dendritically expressed PRG family member, PRG1, has been shown to be located at the postsynaptic density and to be involved in excitatory synaptic transmission (Trimbuch et al., 2009; Unichenko et al., 2016). As synaptic activity is only established during late neurodifferentiation, we asked whether PRG5 is also a postsynaptic density protein, and thus analyzed its subcellular localization. To this end, we performed further subfractionation of mouse neocortex protein lysates, followed by western blot analysis (Cotman and Taylor, 1972; Carlin et al., 1980; Trimbuch et al., 2009). We found PRG5 37 and 150 kDa bands in the myelin-enriched fraction, in the endoplasmic reticulum (ER), in the Golgi-enriched fraction, and in the synaptosomal fraction. In the synaptic cytoplasm and crude synaptic-vesicle fraction, we detected a weak signal for the 150 kDa band, but not for the 37 kDa band. The crude synaptic plasma membrane fraction revealed a strong signal for the 37 kDa band and only a weak signal for the 150 kDa band. Interestingly, we could not detect any PRG5 protein bands in the postsynaptic density (PSD) fraction (Figure 7A).

To further analyze the localization of PRG5 at the synapse, we conducted immunostaining of murine primary hippocampal neurons. We found PRG5 not to be co-localized to marker proteins of the pre- and postsynaptic transmission sites, bassoon and homer1 (Figure 7B, top). Instead, we detected the PRG5 signal between the homer1 signal at the postsynaptic density and MAP2-positive cytoskeletal structures in the dendritic shaft (Figure 7B, bottom). Our results show that PRG5 is located at the neck of dendritic spines, rather than at the actual spine head.

### Plasticity-Related Gene 5 Stimulates the Formation of Dendritic Protrusions Independent From Neuronal Activity

Coiro et al. (2014) showed that down-regulation of PRG5 expression reduces the number of functional synapses in mature hippocampal neurons. To analyze if the PRG5-induced formation of dendritic protrusions in primary hippocampal neurons is depending on synaptic activity, we applied an activity inhibitor cocktail along with PRG5 overexpression. Activity inhibition significantly reduced the number of overall dendritic protrusions, as well as of homer1-positive spines in CFP-MEM transfected control cells (Figure 7C and Supplementary Table 2, line 2). Protrusions between 0.2 and 7 μm lengths were counted and defined as dendritic protrusions, homer1-positive protrusions were defined as spines (Yuste and Denk, 1995; Ruszczycki et al., 2012).

In PRG5 overexpressing neurons, activity inhibition reduced the number of overall protrusions (Figure 7C and Supplementary Table 2, Line 24). Nevertheless, significantly higher numbers of overall protrusions compared to CFP-MEM expressing control cells were found, even in the presence of inhibitors (Figure 7C and Supplementary Table 2, Line 17). A comparable pattern was observed for homer1-positive spines, although the differences were not significant (Figure 7C and Supplementary Table 2, Lines 11, 22, 27). As homer1-positive spines are more likely associated to active synapses, our results indicate activity independent induction of dendritic membrane protrusions and spines by PRG5 overexpression (Figure 7C and Supplementary Table 2).

## Discussion

In this study, we analyzed for the first time the expression and localization of the endogenous PRG5 protein in mouse brain tissue. We identified a PRG5 antibody that specifically detects PRG5 protein in mouse brain tissue samples, in both western blot and immunofluorescence experiments. We confirmed a membranous localization of the endogenous PRG5 in the brain, as well as its N-glycosylation, most likely at the predicted glycosylation site at asparagine 158 (Coiro et al., 2014). In western blot experiments, we identified a band fitting the predicted size of PRG5, with a molecular weight of 37 kDa, but also one very prominent band above 150 kDa. In MS analyses of these bands, isolated from PRG5-eGFP-overexpressing HEK293H cells, we found PRG5 peptides in both bands. Yu et al. (2015) proposed cooperative interactions of PRG family members, including the formation of hetero- and homodimers. Endogenous expression of the neuronal PRGs in HEK293H cells is unlikely, and we did not identify other PRG peptides in our MS analysis. Therefore, we postulate a possible homo-oligomerization of the PRG5 construct, resulting in a 150 kDa protein band. We additionally found this band in mouse brain-tissue samples, but not in liver or spleen samples. Our results indicate a potential homo-oligomerization of the native PRG5 protein in the brain; this needs to be evaluated further.

Using qRT-PCR, PRGs have been shown to be developmentally regulated on the mRNA level (Bräuer et al., 2003; Velmans et al., 2013; Coiro et al., 2014; Gross et al., 2021). We analyzed PRG5-protein expression during cortex development and found a developmental regulation, with increasing expression from late embryonic stages onward. Interestingly, we found an earlier appearance of the band around 150 kDa, whereas the 37 kDa band was only detectable in postnatal stages. This hints at different functional roles for monomeric and oligomeric states of PRGs, which should be considered when analyzing PRG functions.

By immunostaining of adult mouse-brain sections, we found PRG5 protein to be ubiquitously expressed in the brain, but enriched in brain areas that have high plasticity and regeneration capacity, such as the hippocampus or the olfactory bulb. Additionally, we found PRG5 to be predominantly localized to dendritic structures. Previous *in vitro* overexpression studies demonstrated a participation of PRG5 in the formation, morphology, and stabilization of dendritic spines (Coiro et al., 2014). These processes occur in the adult brain in regions in which we found high PRG5 abundance, supporting a similar function for the endogenous PRG5 protein in the brain.

Consistent with previous mRNA-expression analysis in primary cultured brain cells, we found PRG5 protein to be primarily expressed in neurons and in an oligodendrocyte subtype, but not in astrocytes (Coiro et al., 2014; Gross et al., 2021). In neurons, we found PRG5 in both excitatory and inhibitory cells. Another mainly dendritically expressed PRG, PRG1, was shown to be solely expressed by excitatory glutamatergic neurons and to be an important player in LPA-dependent regulation of glutamatergic transmission (Trimbuch et al., 2009; Unichenko et al., 2016). Our mRNA-expression analysis showed high PRG1 expression in adulthood, whereas PRG5 expression increased toward adult stages. Furthermore, we analyzed the subcellular localization of the PRG5 protein and, unlike PRG1, it is not localized at the postsynaptic density, and we could not detect any co-localization with pre- and postsynaptic active-zone proteins at the synapse. In immunostaining, we found PRG5 to be localized at the base of the synapses between the postsynaptic density and the dendritic shaft. Our results suggest that endogenous PRG5 is more likely to play a role in spinogenesis and spine stabilization, than in synaptic transmission processes at the postsynaptic density.

To gain further insight into the time point of PRG5 expression during neurodifferentiation, we used adult neurogenesis as a model system. New-born neurons of the sgz in the hippocampus and the svz pass through distinct developmental stages, as during brain development, and are subsequently integrated into the existing neuronal network (Zhao et al., 2006, 2008). In both areas of adult neurogenesis, we found PRG5 to be only expressed in the postmitotic maturation phase. Stem cells, radial glia, and early neuroblasts did not express the PRG5 protein. Dendritic spine formation and maturation occur during late neurodifferentiation and are associated with a downregulation of DCX, accompanied by an increase of the neuronal marker NeuN (Zhao et al., 2008). We found co-expression of PRG5 and DCX in the MOB, but not in the dentate gyrus. However, co-expression of PRG5 and DCX in the MOB was limited to the late migration phase, where neurodifferentiation proceeds and integration into the neuronal network begins. In both areas, PRG5 was localized to NeuN-expressing neurons. Our results identify PRG5 as a marker for late neurodifferentiation after neuronal fate determination. In a previous study, we demonstrated that knockdown of PRG5 in mature primary hippocampal neurons significantly reduced the number of functional synapses *in vitro* (Coiro et al., 2014). Together with our recent data, this strongly supports a functional role of endogenous PRG5 protein in spinogenesis.

During dendritic differentiation, the shape of dendritic protrusions changes, from thin, elongated filopodia in immature neurons to mature dendritic spines with an enlarged spine head. PRG5 has been shown to induce filopodial outgrowth in non-neuronal cell lines (Broggini et al., 2010; Yu et al., 2015). Our data imply a possible role for PRG5 in shaping dendritic spine morphology. Considering its high expression in mature neurons, and its localization at the bases of synapses, an additional role in dendritic spine stabilization is likely.

Various studies have shown a correlation between neuronal activity and rapid structural as well as functional modifications of dendritic spines (Maletic-Savatic et al., 1999; Chen et al., 2007; Nakahata and Yasuda, 2018). We found PRG5 expressed in areas of the brain characterized by their capability for synaptic plasticity. On a subcellular level, we showed that PRG5 is not associated with the synaptic active zone. Additionally, we showed, that PRG5 significantly induces the formation of dendritic protrusions and tends to induce the formation of homer1-positive spines, when compared to CFP-MEM transfected mature primary cultured neurons. Interestingly, neuronal activity inhibition did not prevent the formation of dendritic membrane protrusions by PRG5 overexpression. Taken together, these data suggest involvement of PRG5 in dendritic spine morphogenesis rather than in synaptic activity.

Many neurological disorders include dendritic spine defects that can affect both spine distribution and spine ultrastructure. The spine pathologies include aberrant number, shape, or localization of spines, as well as subcellular changes of, for example, organelles within the spine (Fiala et al., 2002; Lee et al., 2015). In schizophrenia-spectrum disorders, a reduction of spine number in, for example, the prefrontal cortex or in pyramidal neurons of the hippocampus was seen, whereas an increased number of spines was found in the putamen patch and the caudate matrix (Roberts et al., 1996, 2005; Garey et al., 1998; Glantz and Lewis, 2000; Kolomeets et al., 2005; Sweet et al., 2009). Genes and proteins that have been linked to the regulation of spine morphology and development, such as Neuregulin-1 and ErbB4 or Cdc42, show aberrant expression or signaling in schizophrenia patients (Stefansson et al., 2002; Hashimoto et al., 2004; Hill et al., 2006; Pan et al., 2011). Functional analysis suggests that PRG5 acts through a Cdc42-independent pathway to promote the formation of membrane protrusions (Broggini et al., 2010). Understanding the complex mechanisms of initial spine formation and their stabilization and regulation mechanisms is of vital importance, including for therapeutic research on neurological disorders. Considering its high expression during late neurodifferentiation, PRG5 is likely to be an important player in these processes, acting via an unidentified signaling pathway that could be of interest in therapeutic approaches to spine pathologies.

## Conclusion

We found the endogenous PRG5 protein in mouse brain tissues in its monomeric 37 kDa state, as well as in a larger 150 kDa state, most likely in a homo-oligomeric structure. In mouse brain slices, we found ubiquitous expression of PRG5 in soma and dendrites of neurons, with high abundance in the hippocampus, cerebellum, and the olfactory bulb. PRG5 expression in neurons is independent of their neurotransmitters, and we found PRG5 in an oligodendrocyte subtype, but not in astrocytes. In adult neurogenesis, PRG5 is only expressed during a late phase of neurodifferentiation, associated with spinogenesis. On a subcellular level, we localized PRG5 to the bases of dendritic spines, rather than to the postsynaptic density. Additionally, PRG5-induced formation of dendritic protrusions is independent from neuronal activity. Therefore, our results support an important participation of PRG5 in dendritic spine morphogenesis.

## Experimental Procedures

### Animals

Time-pregnant, postnatal, and adult wild-type C57BL/6J mice were procured from the central animal facility of the University Medical Center Rostock or the central animal facility of the Carl von Ossietzky University of Oldenburg. They were kept under standard laboratory conditions in accordance with German and European guidelines for the use of laboratory animals (2010/63/EU; 12 h light/dark cycle; 55% ± 15% humidity; 22°C ± 2°C room temperature (RT) and water *ad libitum*, enriched and grouped). All protocols were in accordance with the German Animal Protection Law and were approved by the local ethics body of Mecklenburg-Western Pomerania (LALLF) and Lower Saxony (LAVES; TV 33.19-42502-04-18/2759 and 33.19-42502-04-18/2766). For determining embryonic stages, the day of the vaginal plug following mating was assigned as embryonic day 0.5 (E0.5). Mice of both sexes were used for protein lysis and immunostaining.

### HEK293H Cell Culture and Transfection

HEK293H cells (Thermo Fisher Scientific, Waltham, MA, United States; Catalog ID 11631017) were routinely maintained in Dulbecco’s Modified Eagle Medium (DMEM; Thermo Fisher Scientific) supplemented with 10% fetal bovine serum (FBS; PAN-Biotech, Aidenbach, Germany), 2 mM L-glutamine (Merck Millipore, Darmstadt, Germany), 100 units/ml penicillin, and 100 μg/ml streptomycin (PAN-Biotech) under standard conditions of 5% (v/v) CO_2_ and 37°C. Cells were passaged every 3–4 days at around 90% confluency. For transfection, cells were seeded in 100 mm petri dishes with 1.5 × 10^6^ cells per dish and transfected after 24 h with calcium-phosphate precipitation. For this purpose, 22 μg DNA in 337.5 μl sterile H_2_O and 37.5 μl 2.5 M CaCl_2_, was precipitated by slow drop-by-drop addition of 337 μl HEPES-buffered saline pH 7.05 (42 mM HEPES, 10 mM KCl, 1.5 mM Na_2_HPO_4_, 1.1 mM glucose), then incubated for 30 min at RT and added to the cells. Cells were lysed after 24–48 h. The following expression plasmids were used for transfection: peGFP-N1-mPRG1, peGFP-N1-rPRG2, peGFP-N1-mPRG3, peGFP-C1-mPRG4, peGFP-N1-rPRG5 (Savaskan et al., 2004; Broggini et al., 2010; Velmans et al., 2013; Coiro et al., 2014).

### Tissue and Cell Protein Lysis, Purification, and Deglycosylation

For obtaining protein lysates of different organs and brain areas, mice of varied developmental stages were sacrificed by cervical dislocation, individual organs were removed, and specific brain areas dissected on ice. For embryonic stages, tissue samples of all embryos of one litter (seven to ten embryos; sex not specified) were pooled. Postnatal tissue samples were pooled from four to eight mice of both sexes. For cortex samples, three pooled samples from three independent preparations were used. For other brain areas, one pooled sample of one preparation was used (Supplementary Figure 2A). Liver and spleen samples for antibody verification were obtained from a single adult female mouse.

Cortex tissue was then homogenized with a syringe and cannula in lysis buffer containing 20 mM Tris-HCl pH 7.5, 250 mM sucrose, 1 mM EGTA, 5 mM EDTA, 1x cOmplete mini protease inhibitor cocktail (Sigma-Aldrich, St. Louis, MO, United States) and 1x PhosSTOP phosphatase inhibitor (Sigma-Aldrich). Cultured cells were washed twice with ice-cold 1x PBS, lysis buffer was added, and cells were scraped off the culture plates with a cell scraper. All samples were sonicated for 3 × 3 s and then incubated on ice for 30 min. Cell debris was pelleted by centrifugation at 10,000–12,000 × g for 10–15 min at 4°C and total protein lysates were stored for the short term at –20°C or the long term at –80°C. For separation of cytosolic and membrane protein fractions, total protein lysates were further centrifuged at 200,000 × g for 30 min at 4°C. The supernatant was collected as the cytosolic protein fraction, while the pellet was re-suspended in lysis buffer additionally containing 1% (v/v) Triton X-100, and incubated for 10 min on ice. Samples were again centrifuged for 10 min at 4°C and 100,000 × g; the resulting supernatant contained fractions of membrane proteins. Protein concentrations were determined by BCA protein assay (Thermo Fisher Scientific) with BSA standards, following the manufacturer’s protocol.

For purification of eGFP fusion proteins, lysates of transfected HEK293H cells were processed with the μMACS GFP Isolation Kit (Miltenyi Biotec, Bergisch Gladbach, Germany) according to the manufacturer’s instructions.

For the deglycosylation assay, P15-cortex protein lysates were treated with Peptide-N-glycosidase F (PNGase F, Roche, Basel, Switzerland) as recommended by the manufacturer, run over night at 37°C. Negative controls were incubated without PNGase F.

### Western Blot

Protein lysates were diluted in 5 × Laemmli buffer containing 1% (v/v) 2-mercaptoethanol or, for PRG5 detection, without 2-mercaptoethanol but with the addition of 8 M urea. Proteins were separated by SDS-PAGE and transferred on nitrocellulose (NC) membranes with tank-blotting procedure for 70 min at 100 V and 4°C. To establish the total protein content, blotted membranes were briefly stained with 0.1% (w/v) Ponceau-S in 5% (v/v) acetic acid. Non-specific antibody binding was blocked with 5% (w/v) bovine serum albumin (BSA; Carl Roth, Karlsruhe, Germany) or 2.5–5% milk (Carl Roth) in PBS or TBST. Primary antibody incubation was carried out in blocking solution overnight at 4°C (for antibodies and concentrations, see Table 1).

**TABLE 1:** Primary antibodies and concentrations used for immunochemistry and western blot experiments.

Antibody	Dilution for western blot	Dilution for immunochemistry	Source	Identifier
Anti-bassoon	–	1:100	Synaptic Systems	Cat# 141011, RRID: AB_2619827
Anti-doublecortin	–	1:2,000	Merck Millipore	Cat# AB2253, RRID: AB_1586992
Anti-GFAP	–	1:500	Merck Millipore	Cat# MAB360, RRID: AB_11212597
Anti-GFP (JL-8)	1:2,500	–	Clontech Laboratories	Cat# 632380, RRID: AB_10013427
Anti-GFP		1:1,000	Abcam	Cat# ab6556, RRID:AB_305564
Anti-homer 1	–	1:200	Synaptic Systems	Cat# 160 004, RRID: AB_10549720
Anti-HSP60 (LK-2)	1:2,000	–	Enzo Life Science	Cat# ADI-SPA-807-E, RRID: AB_2039242
Anti-MAP2 (HM-2)	–	1:1,000	Sigma-Aldrich	Cat# M4403, RRID: AB_477193
Anti-MAP2	–	1:1,000	Synaptic Systems	Cat# 188 004, RRID: AB_2138181
Anti-MBP (12)	1:500	–	Merck Millipore	Cat# MAB386, RRID: AB_94975
Anti-Nestin (rat-401)	–	1:100	Merck Millipore	Cat# MAB353, RRID: AB_94911
Anti-NeuN (1B7)	–	1:1,000	Abcam	Cat# ab104224, RRID: AB_10711040
Anti-Neurofilament M (NN18)	–	1:200	Merck Millipore	Cat# MAB5254, RRID: AB_2150076
Anti-Parvalbumin	–	1:1,000	Sigma-Aldrich	Cat# P3088, RRID: AB_477329
Anti-PRG5	1:500	1:300	Abcam	Cat# ab84659, RRID: AB_1925293
Anti-PSD95 (7E3-1B8)	1:1,000	1:500	Thermo Fisher Scientific	Cat# MA1-046, RRID: AB_2092361
Anti-sodium potassium ATPase (464.6)	1:200	–	Abcam	Cat# ab7671, RRID: AB_306023
Anti-synaptophysin 1	1:500	–	Synaptic Systems	Cat# 101 011, RRID: AB_887824
Anti-TH (Inc1)	–	1:200	Merck Millipore	Cat# MAB318, RRID: AB_2201528
Anti-α-tubulin	1:3,000	–	Synaptic Systems	Cat# 302 211, RRID: AB_887862

Membranes were washed three to six times at RT with PBST or TBST, and subsequently incubated with horseradish peroxidase-conjugated secondary antibodies (GE Healthcare, Boston, MA, United States) 1: 10,000 in 2.5% (w/v) BSA in PBST or TBST for 1.5 h at RT. Membranes were again washed 3 times at RT with PBST, TBS or TBST, and any immunoreaction was detected with Clarity or Clarity Max ECL substrate (Bio-Rad Laboratories, Hercules, CA, United States) according to the manufacturer’s protocol.

### Immunohistochemistry

Adult mice were deeply anesthetized with sodium pentobarbital (Narcoren; Boehringer Ingelheim, Germany), then transcardially perfused with cold 1x PB, followed by cold 4% (w/v) paraformaldehyde (PFA; Merck Millipore) in 1x PB fixation solution. P0 mice were sacrificed by cervical dislocation. Brains were removed and additionally immersion-fixed in 4% (w/v) PFA in 1x PB for 24–48 h at 4°C. Coronal or sagittal 35–40 μm brain sections were prepared on a Leica VT1000S vibratome (Leica Microsystems, Wetzlar, Germany). To reduce autofluorescence, slices were treated with 1% (v/v) NaBH_4_ in 0.1 M PB pH 7.2 for 15–60 min at RT, with mild agitation. Slices were washed four times for 5 min with PB, followed by blocking of non-specific antibody binding overnight at 4°C with 10% (v/v) FBS or 1% BSA, 1% (v/v) normal goat serum (NGS; Vector laboratories, Burlingame, CA, United States), and 0.2% (w/v) saponin in PB. Primary antibodies were diluted in 5% (v/v) FBS or 0.5% BSA, 1% (v/v) NGS and 0.2% (w/v) saponin, and slices were incubated for 48 h at 4°C, with gentle agitation. Primary antibodies and concentrations are listed in Table 1. Slices were washed three times with PB with 0.2% (w/v) saponin for 10, 20, and 30 min at RT. Secondary-antibody and DAPI incubation was performed overnight at 4°C in the same solution as primary-antibody incubation. The following secondary antibodies were used: goat-anti-mouse IgG Alexa Fluor 568 (1:1,500; Thermo Fisher Scientific), goat-anti-rabbit IgG Alexa Fluor 488 (1:1,500; Thermo Fisher Scientific) and goat-anti-guinea pig Cy3 (1:400; Jackson Immuno Research, West Grove, PA, United States). DAPI (Carl Roth) was used at 0.5 μg/ml, and added to the secondary-antibody solution. Slices were again washed three times at RT with 0.1 M PB, mounted on microscopy glass slides, and dried for 20 min at RT. For further microscopic analysis, slices were cover-slipped with Mowiol/DABCO (Carl Roth) or Immumount (Thermo Fisher Scientific). Nomenclature of brain regions was taken from The Allen Mouse Brain Atlas (RRID:SCR_002978) and The Allen Developing Mouse Brain Atlas (RRID:SCR_002990). All immunostainings were performed at least three times with sections from at least three different animals with different sexes. Stainings revealed similar results for all animals and sexes and representative images were chosen for this publication.

### Primary Mouse Hippocampal Neuron Culture

Hippocampi of all E18 (± 0.5 days) mouse embryos from one pregnant mouse were dissected and primary neuron cultures prepared as previously described by Brewer et al. (1993). Neurons were plated onto poly-l-lysine-coated glass coverslips (Sigma-Aldrich) at a density of 2.1 × 104 cells/cm^2^ in Minimal Essential Medium (Thermo Fisher Scientific) supplemented with 0.6% glucose, 10% (v/v) horse serum, and 100 U/ml penicillin/streptomycin (PAN-Biotech). Media were changed after 3–4 h to Neurobasal A media, supplemented with 2% (v/v) B27, 0.5 mM glutamine (all from Gibco, Thermo Fisher Scientific) and 100 U/ml penicillin/streptomycin.

Primary cultured neurons were transfected at DIV 14 with Effectene (Qiagen, Hilden, Germany) according to manufacturer’s instructions. Media was changed 3 h after transfection. The following expression plasmids were used for transfection: pEGFP-N1-rPRG5 (Coiro et al., 2014) and pECFP-MEM (Takara Bio Inc., Clontech, Kusatsu, Japan).

Primary neurons were treated with an activity inhibitor cocktail or solvents 3 h after transfection along with change of media after transfection. Cells were incubated for 20 h. Activity inhibitor cocktail contained 50 μm D(–)-2-amino-5-phosphonopentanoic acid (D-APV) (in ddH_2_O; Sigma-Aldrich, A8054), 10 μM nifedipine (in DMSO; Research Biochemicals International, N-114, Natick, MA, United States) and 1 μM tetrodotoxin (TTX) (in ddH_2_O; Alomone Labs, T-550, Jerusalem, Israel) (Yu and Malenka, 2003; Brandt et al., 2007; Xie et al., 2007).

For immunocytochemical staining, neurons on coverslips were fixed with 4% (w/v) PFA and 15% (w/v) sucrose in PBS for 15 min at RT. For stainings including the PRG5 antibody, coverslips were washed three times with PBS and cells were blocked with 5% (v/v) NGS and 0.2% (w/v) saponin in PBS for 1 h at RT. Primary antibody incubation was carried out overnight at 4°C in 1% (v/v) NGS and 0.2% (w/v) saponin in PBS; primary antibodies and concentrations are listed in Table 1. Cells were washed three times for 10 min with PBS at RT. Secondary antibodies were incubated for 90 min at RT in the same solution as primary antibodies. For stainings after activity inhibition, cells were fixed as described. Permeabilization was carried out 20 min at RT with 0.5% Triton-X-100 and 0.1% sodium-citrate in PBS. Cells were wahsed three times with PBS and blocked for 1 h at RT with 10% FBS and 1% NGS in PBS. Antibody incubation was carried out in 5% FBS and 1% NGS overnight at 4°C for primary and for 90 min at RT for secondary antibodies. The following secondary antibodies were used: goat-anti-rabbit IgG Alexa Fluor 488 (1:1,500; Thermo Fisher Scientific), goat-anti-guinea pig Cy3 (1:400; Jackson Immuno Research), goat-anti-mouse IgG Alexa Fluor 647 (1:1,500; Thermo Fisher Scientific) and DAPI (0.5 μm/μl). Coverslips were washed three times with PBS and mounted on microscope slides with Mowiol/DABCO.

### Microscopy

Fluorescent images of brain slices were obtained using an IX83 inverted-imaging system with a DP80 camera and 10x, 20x, or 40x UPlanSApo (0.75 NA) and 60x UPlanSApo oil-immersion (1.35 NA) objectives (Olympus, Shinjuku, Japan).

For confocal-image acquisition, either an upright TCS SP8 Laser microscope equipped with a 63x objective (oil-immersion, 1.2 NA) using sequential scanning with the 488 nm line of an argon-ion laser and the 543 nm line from helium–neon lasers (for Alexa 488 and Alexa 568, respectively) (Leica Microsystems) or an inverted confocal laser scanning FV3000 microscope equipped with 10x (0.75 NA), 20x (0.8 NA), and 40x (oil-immersed, 1.4 NA) objectives using sequential scanning with 405 nm, 488 nm, 561 nm cw diode lasers (for DAPI, Alexa488, Cy3 or Alexa 568, respectively) (Olympus) was used. Background correction and adjustment of brightness and contrast were performed using either LasX confocal software version 3.5.7 (Leica Microsystems, RRID:SCR_013673), cellSens Dimension version 1.18 (Olympus) or Fiji (NIH, Bethesda, MD, RRID:SCR_002285). Image analysis of protrusions was performed using cellSens Software (Olympus).

### Subcellular Fractionation of Mouse Total-Brain Protein Lysates

Subcellular fractionation was adapted from previously described protocols (Cotman and Taylor, 1972; Mizoguchi et al., 1989; Trimbuch et al., 2009). Adult mice were sacrificed and brains dissected as described. Brains were homogenized in 0.32 M sucrose, 1 mM NaHCO_3_ in H_2_O with a syringe and cannula. Homogenate was layered on top of a sucrose gradient consisting of 0.85, 1, and 1.2 M sucrose solutions and ultra-centrifuged at 82,000 × g, at 4°C for 2 h (Beckmann Coulter, Brea, CA, United States). After centrifugation, the topmost layer of 0.32 M sucrose contained the cytosolic fraction, the band between 0.32 and 0.85 M sucrose contained the myelin-enriched fraction, the band between 0.85 and 1 M sucrose contained the Golgi- and ER-enriched fraction, the band between 1 and 1.2 M sucrose contained the synaptosomal fraction, and the pellet consisted of the mitochondria-enriched fraction. The synaptosomal fraction was further centrifuged at 33,000 × g at 4°C for 20 min and the resulting pellet was re-suspended in 6 mM Tris/HCl pH 8.0 for hypo-osmotic shock. The solution was again centrifuged at 33,000 × g at 4°C for 20 min. The resulting supernatant contained synaptic cytoplasm and the crude synaptic-vesicle fraction. The crude synaptic-plasma membrane fraction was extracted by re-suspending the pellet with 6 mM Tris/HCl pH 8 and 1% (v/v) Triton X-100. The crude synaptic plasma membrane fraction was then centrifuged a 33,000 × g at 4°C for 10 min; the resulting pellet contained the postsynaptic density fraction.

### Proteomic Analysis

Following electrophoresis, gels were stained with Coomassie Brilliant Blue (Neuhoff et al., 1988) and slices of interest excised from the gels. Each slice was cut into small pieces (∼1 mm^2^) prior to washing, reduction, alkylation and tryptic digestion (Kossmehl et al., 2013). The resulting peptides were separated applying nano-liquid chromatography using a trap column (2 cm, C18, 5 μm bead size, 75 μm inner diameter; Thermo Fisher Scientific) coupled to a 25 cm analytical column (C18, 2 μm bead size, 75 μm inner diameter; Thermo Fisher Scientific) using a 180 min linear gradient (Wöhlbrand et al., 2016). Eluting peptides were ionized online (captive spray ion source, Bruker Daltonik GmbH, Bremen, Germany) and mass analyzed by an ion-trap mass spectrometer (amaZon speed ETD, Bruker Daltonik). Positive ions were analyzed with a capillary current of 1.3 kV and dry-gas flow of 3 l/min nitrogen at 150°C. Active precursor exclusion was set for 0.2 min and 20 MS/MS spectra per full scan MS acquired. Protein identification was performed by Mascot (version 2.3, Matrix Science Ltd., London, United Kingdom) operated via the ProteinScape platform (Version 4.2, Bruker Daltonik GmbH) searching against the translated genome sequences of *Mus musculus*. Search settings were as follows: significance threshold *p* < 0.05; mass tolerance MS 0.3 Da, MS/MS 0.4 Da; false discovery rate 1.0% (applying target decoy); 1 missed cleavage site allowed; oxidation M variable modification; carbamidomethyl C fixed modification. A summary of identified proteins is provided in Supplementary Table 1.

### Data Analysis

Data analysis was performed using GraphPad Prism 7.05 (GraphPad Software Inc., La Jolla, CA, United States). All data are presented as mean + SEM. A level of confidence of *p* ≤ 0.05 was adopted [*p ≤ 0.05, ^**^p ≤ 0.01, ^***^p ≤ 0.001, ns = not significant (*p* > 0.05)]. Values were analyzed for normal distribution using the D’Agostino and Pearson test. Statistical analysis was performed using the 1 way ANOVA (robust against violation of normal distribution; Schmider et al., 2010).

## Data Availability Statement

The original contributions presented in the study are included in the article/Supplementary Material, further inquiries can be directed to the corresponding author/s.

## Ethics Statement

The animal study was reviewed and approved by the Landesamt für Landwirtschaft, Lebensmittelsicherheit und Fischerei Mecklenburg-Vorpommern (LALLF) and Niedersächsisches Landesamt für Verbraucherschutz und Lebensmittelsicherheit (LAVES).

## Author Contributions

AB, IG, and NB designed the study. AB and IG wrote the manuscript, with contribution from all co-authors. IG performed western blot experiments and immunostainings and analyzed the results. DV performed primary neuron transfection and immunostainings. FK performed western blot experiments. NB performed and analyzed activity inhibitor experiments. LW and RR performed the proteomic analysis. MW helped with RMS analysis. AH, TP, and MH revised the manuscript. Data from this work was part of the Ph.D. theses of IG, DV, and FK. All authors read and approved the final manuscript.

## Conflict of Interest

The authors declare that the research was conducted in the absence of any commercial or financial relationships that could be construed as a potential conflict of interest.

## Publisher’s Note

All claims expressed in this article are solely those of the authors and do not necessarily represent those of their affiliated organizations, or those of the publisher, the editors and the reviewers. Any product that may be evaluated in this article, or claim that may be made by its manufacturer, is not guaranteed or endorsed by the publisher.
