# Supplementary material for: Plasticity-Related Gene 5 Is Expressed in a Late Phase of Neurodifferentiation After Neuronal Cell-Fate Determination
Source: Front Cell Neurosci. 2022 Apr 15;16:797588. doi: 10.3389/fncel.2022.797588 (PMC9053830; doi:10.3389/fncel.2022.797588)

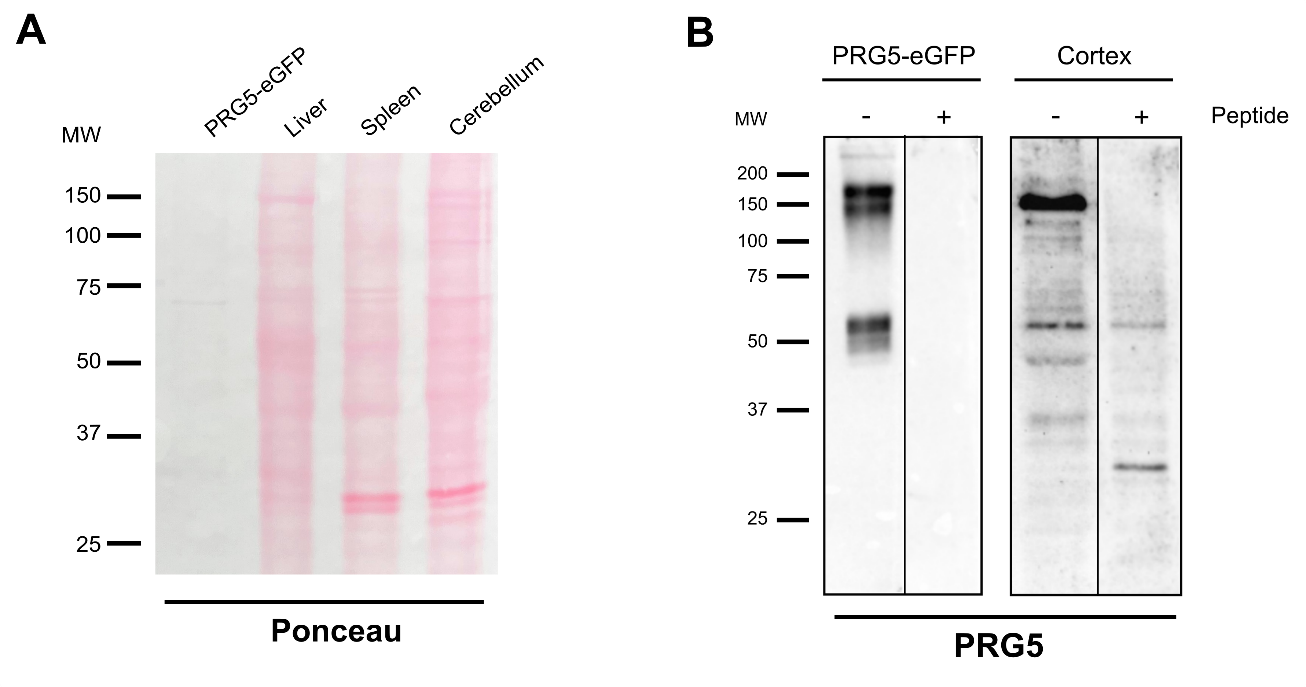


**Figure S1. (A)** Ponceau-S staining of total proteins as a loading control for western blot of Fig. 1B in the main manuscript.


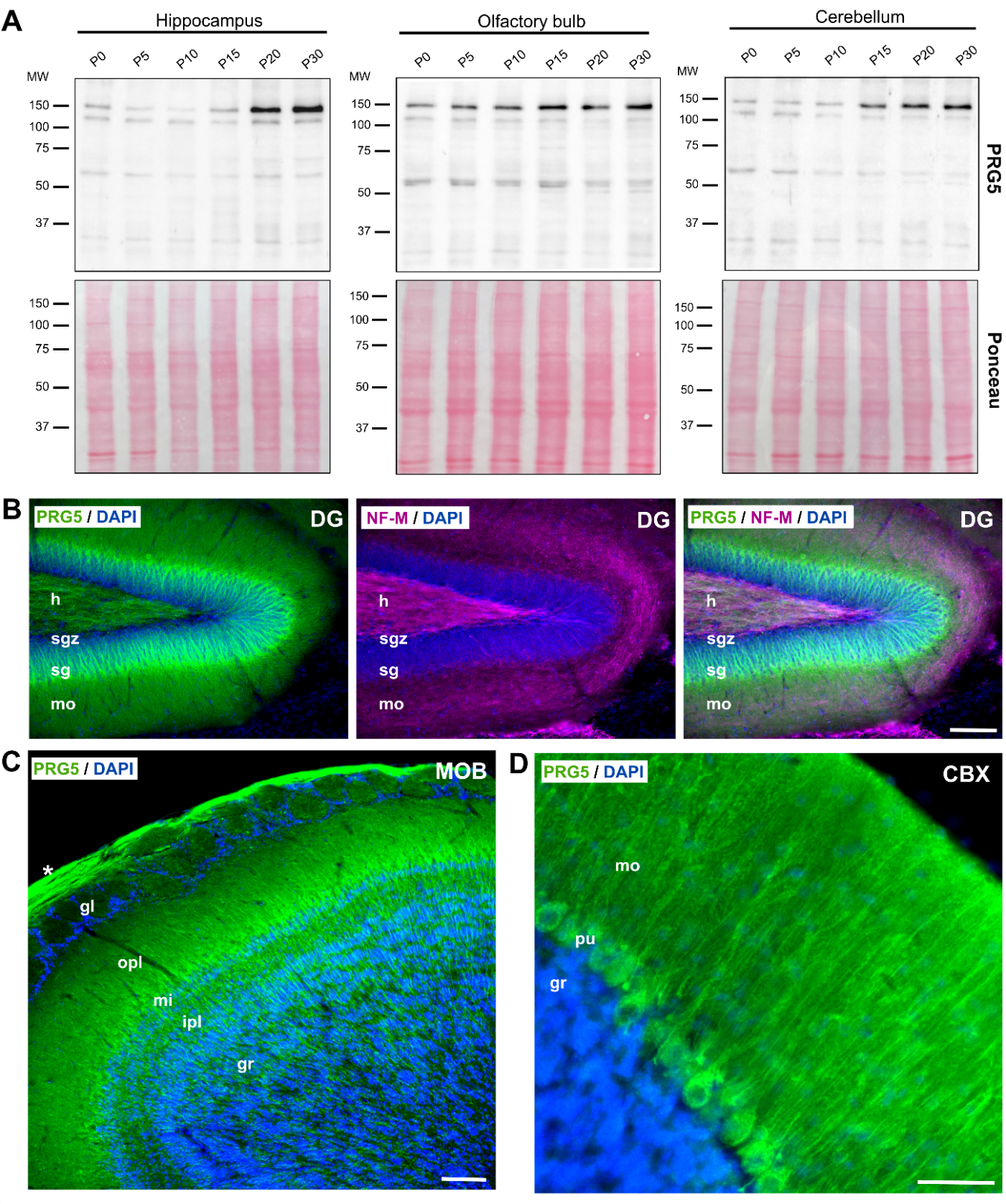


**Figure S2. PRG5 protein expression during brain development and in the adult brain. (A)** Representative western blot analysis of total protein lysates of mouse brain tissue samples between P0 and P30, probed with an anti-PRG5 antibody. Ponceau-S staining of total proteins is shown as a loading control. A representative blot of three technical repeats for each of three tissue preparations is shown. **(B)** Low axonal expression of PRG5 (green) in the DG of the hippocampus is shown by co-immunostaining with axonal marker NF-M (magenta). Strong PRG5 expression was found in granule cells in the sg and their dendritic arbors in the mo, whereas there was only low expression in their mossy fibers in the hilum. Scalebar = 100 µm. **(C)** High PRG5 expression in the MOB, especially in the ipl and opl and in mitral cells (mi). Scale bar = 100 μm. **(D)** Purkinje cells of the CBX showed strong PRG5 signal in their soma and in their dendritic arbors in the mo. Scale bar = 50 μm. DAPI visualizes cell nuclei (blue). CA,cornu ammonis; CBX, cerebellar cortex; DG, dentate gyrus; gl, glomerular layer; gr, granule layer; h, hilus; ipl, inner plexiform layer; mi, mitral layer; mo, molecular layer; MOB, main olfactory bulb; opl, outer plexiform layer; pu, Purkinje layer; sg, stratum granulosum; sgz, subgranular zone; slm, stratum lacunosum-moleculare; so, stratum oriens; sp, stratum pyramidale; sr, stratum radiale.. Representative images; Stainings of brain sections from at least three different animals were analyzed and revealed similar results.

**Table S2:** Statistics Fig. 7C; 1way ANOVA (*p≤0.05, **p≤0.01, ***p≤0.001, ns = not significant [p>0.05]). followed by Tukey’s multiple comparison test; Activity Inhibitor (Act Inh); vs = versus.


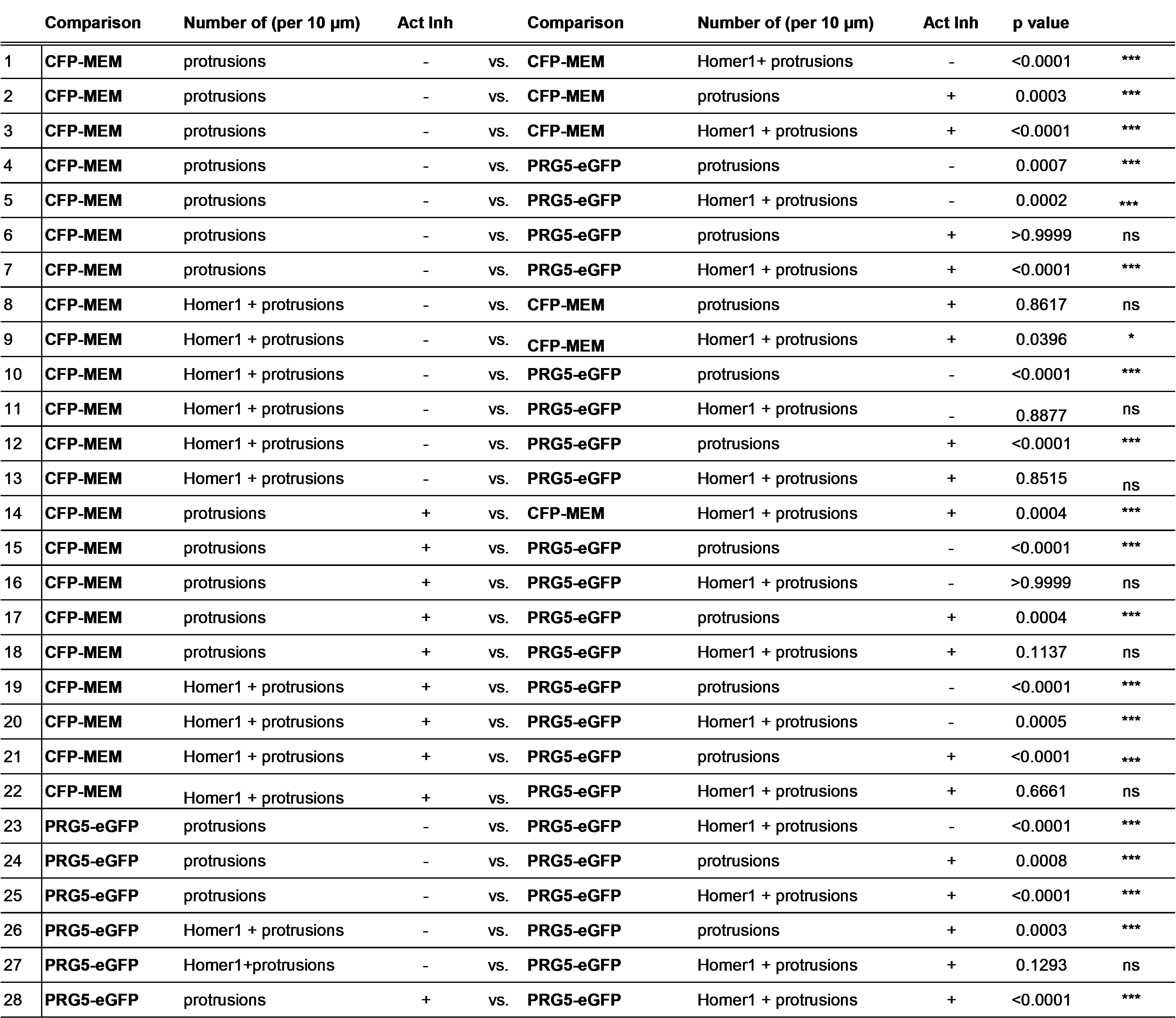

Supplement: Supplementary file 2 [file Data_Sheet_1.docx]
